# Postnatal care utilization among urban women in northern Ethiopia: cross-sectional survey

**DOI:** 10.1186/s12905-018-0557-5

**Published:** 2018-05-30

**Authors:** Genet Gebrehiwot, Araya Abrha Medhanyie, Gebreamlak Gidey, Kidan Abrha

**Affiliations:** 1 Mekelle Hospital, Mekelle, Tigrai Ethiopia; 2 School of Public Health, College of Health Sciences, Mekelle University, Mekelle, Ethiopia; 3 Department of Midwifery, College of Health Sciences, Aksum University, Aksum, Ethiopia

**Keywords:** Postnatal care, Utilization, Mekelle, Tigray, Ethiopia

## Abstract

**Background:**

Postnatal care service enables health professionals to identify post-delivery problems including potential complications for the mother with her baby and to provide treatments promptly. In Ethiopia, postnatal care service is made accessible to all women for free however the utilization of the service is very low. This study assessed the utilization of postnatal care services of urban women and the factors associated in public health facilities in Mekelle city, Tigrai Region, Northern Ethiopia.

**Methods:**

A facility based cross sectional study design was used to assess post natal service utilization. Using simple random sampling 367 women who visited maternal and child health clinics in Mekelle city for postnatal care services during January 27 to April 2014 were selected. Data was entered and analyzed using SPSS Version 20.0 software. A binary and multivariable logistic regression was used to identify risk factors associated with the outcome variables. *P*-value less than 0.05 is used to declare statistical significance.

**Results:**

The prevalence of women who utilized postnatal care service was low (32.2%). Women who were private employees and business women were more likely to utilize postnatal care services (AOR = 6.46, 95% CI: 1.91–21.86) and (3.35, 95% CI: 1.10–10.19) respectively compared to house wives., Women who had history of one pregnancy were more likely to utilize the service (AOR = 3.19, 95% CI: 1.06–9.57) compared to women who had history of four and above pregnancies. Women who had knowledge of postnatal care service were also more likely to utilize postnatal care service (AOR = 14.46, 95% CI: 7.55–27.75) than women who lacked knowledge about the services**.**

**Conclusions:**

Postnatal care utilization in the study area is low. Knowledge on postnatal care services and occupation of women had positive impact on postnatal care service utilization. The Mekelle city administration health office and other stakeholders should support and encourage urban health extension workers and health facilities to strengthen providing health education to improve the knowledge of the women about the importance of postnatal care services.

**Electronic supplementary material:**

The online version of this article (10.1186/s12905-018-0557-5) contains supplementary material, which is available to authorized users.

## Background

Ethiopia is one of the countries in Sub Saharan Africa (SSA) with markedly high maternal and neonatal mortality ratio and it was estimated at 676 maternal deaths per 100,000 live births and neonatal mortality rate 37 deaths per 1000 live births in 2011 [1]. Neonatal death accounted for 62% of all infant deaths and 44% of all under-five deaths [1, 2].

Ethiopia has been implementing high impact and cost-effective health interventions as well as strengthening its health system to improve the health status of its population and reduce maternal and neonatal mortality. These interventions include scale up of family planning programs, training and deployment of more midwives, referral system including pediatric referral system, service integration, health extension program (HEP), routine immunization and wild polio reduction. And yet maternal and neonatal mortality rates remain high [3].

The common medical causes for maternal deaths include bleeding, high blood pressure, prolonged and obstructed labour, infections and unsafe abortion [4]. Bleeding and infection following childbirth account for many maternal deaths [5]. Hemorrhage and sepsis accounted for 27.1 and 10.7% of maternal mortality respectively [6] while preterm birth, asphyxia and severe infections contributed to two thirds of all neonatal death [4, 5].

The postnatal period begins immediately after the birth of the baby and extends up to 6 weeks (42 days) after birth. The major purpose of postpartum and postnatal care is to maintain and promote the health of the woman and her baby and to foster an environment that offers help and support to the extended family and community for a wide range of related health and social needs [1, 5].

Two thirds of maternal and newborn deaths occur in the first 2 days after birth [5]. Hence, having postnatal care is important for both the mother and the child to avoid the risk of preventable death by treating complications arising from the delivery as well as to provide the mother with important information on how to care for herself and her child [2].The World Health Organization (WHO) recommends postnatal visits within the first 24 h from birth, on day 3 (48–72 h) and between days 7–14 after birth, and 6 weeks after birth [7].

Postpartum care for the mother has conventionally focused on routine observation and examination of vaginal blood loss, uterine involution, blood pressure and body temperature [5]. Similarly, postnatal care for all newborns should include immediate and exclusive breastfeeding, warming of the infant, hygienic care of the umbilical cord, and timely identification of danger signs with referral and treatment [8]. Postnatal care is the routine care services that every woman and her baby should be offered, appropriate to their individual circumstances after the birth of the baby until the conclusion of the postnatal period [9]. In the absence of postnatal follow-up, numerous cases of puerperal infections become undiagnosed and unreported [10]. Lack of care in postnatal period from skilled providers may result in death or disability as well as missed opportunities to promote healthy behaviors affecting women, newborns, and children [11].

Although PNC has several benefits and reduces maternal and child mortality significantly, postnatal service use is low in most of SSA countries [12]; In Ethiopia PNC services are made accessible to all women for free, however, the utilization of the services is very low [13]. The findings of the three Ethiopian demographic health surveys (EDHS) showed an improvement in ANC utilization from 27% in 2000 to 28% in 2005 and to 34% in 2011 However, utilization of PNC remained below 10% (3.4% in 2000, 5.8% in 2005 and 7% in 2011) [1]. The EDHS 2011 showed that 32% of women in urban areas received a postnatal checkup from a health professional compared to 2% in rural women [4].

There were few community based studies conducted in Ethiopia which depicted the magnitude of PNC service utilization and associated factors. There has been no facility based studies which showed the magnitude of PNC service utilization and associated factors among urban women. This study addresses the extent and factors affecting PNC service utilization among women who visited health facilities for any maternal and child health services in an urban setting.

## Methods

### Study design and area

A facility based cross-sectional study was conducted from January to April 2014 among all women who had a child aged 45 days up to 6 months and visited maternal and child health (MCH) clinic in selected public health facilities in Mekelle city, Tigrai regional state, Ethiopia. Mekelle is the capital city of Tigrai regional state. The city has 7 sub administrative cities, 3 government hospitals and 9 public health centers providing MCH services. According to the Mekelle zonal health bureau profile, the total population of the city in 2013 was estimated to be 301,642 of which 147,804 were male and 153,837 female. Women in the reproductive age group (15–45) were 70,825 while children under 1 year were estimated to be 10,747 [14]. Women who were critically ill, women who had mental health problems, and those unable to provide informed consent or women with critically ill children were excluded from the study..

### Sample size and sampling technique

A total sample size of 367 was determined using single proportion formula. When calculating the sample size; it was assumed that a 95% confidence interval, 32% of postnatal care utilization (taken from EDHS 2011), 5% marginal error and 10% contingency.

Of the health facilities in the city (2 hospitals and 9 health centers), seven (2 hospitals and 5 health centers) were selected. The selection of the health facilities was done randomly. The health facilities were further stratified by patient flow of the previous 3 months prior to data collection and considering the average client flow at each health facility. The total sample size determined (367) was distributed proportionally to patient flow size among the selected health facilities.

To select each woman from a selected health facility, systematic random sampling was employed. The sampling interval (*K*^*th*^) among each study subject was calculated by dividing the study population of each selected health facility to the sample size deployed to each selected health facility. The first study subject was selected randomly from the range of (1 up to *K*^*th*^) and thereafter every participant was selected every *K*^*th*^ interval until the sample size was obtained for all the selected health facilities.

### Data collection procedures

A structured questionnaire was used to interview women (Additional file 1). The questionnaire was adapted by reviewing relevant literature and questionnaire such as EDHS 2011 and WHO guideline for PNC. The questionnaire was prepared first in the English language and translated into Tigrigna, the local language and then translated back to English by people proficient in both languages to maintain the consistency and content of the questionnaire.

Three data collectors who completed high school were recruited. The data collectors were given a 1 day training on the objectives and relevance of the study: the contents of the training were:, on how to ensure confidentiality of information, understand the meanings of each question in the questionnaire, how to approach participants, how to follow ethical procedures and general information on postnatal care follow up.

A supervisor with a Bachelor of Science in midwifery was also recruited for the data collection. The supervisor was responsible in the supervision and support of data collectors, checking filled out questionnaire for completeness daily and providing feedback for data collectors. In addition to the supervisor, the principal investigator (GG) is participated in the supervision of data collection.

The Tigrigna version of the questionnaire was pretested among 18 women (5% of the total sample size) who had visited MCH clinic. This pre-test was done in health facilities which were not selected for this study.

### Data processing and analysis

Data was entered into a computer using SPSS window version 20.0 and cleaned. Descriptive statistics was employed to calculate frequencies and display findings. Association was measured using binary logistic regression. Based on bivariate analysis, variables that showed significant association at (*p* < 0.05) were entered to multivariable analysis to select predictor variables of PNC service utilization.

The final model was built by using enter method standard regression model building technique. Before building the final model, multi co linearity effect was assessed using linear regression and the mean VIF > 5 was used as cut off point. The final model was then tested for its goodness of fit by Hosmer and Lemeshow *p*-value and *p* value > 0.05 was best fit. Finally, variables that showed significant association at (*P* < 0.05) were identified as independent predictors of PNC service utilization.

## Result

### Socio demographic characteristics of women

A total of 367 women participated in the study making a response rate of 100%. One hundred twenty nine (35.1%) of the respondents came for immunization followed by 82(22.3%) care of sick baby, 78 (21.3%) family planning, 37 (10.1%) care of sick mother, 34 (9.3%) both immunization of baby and family planning and 7(1.9%) came for circumcision of baby (Table 1).

**Table 1:** Socio-demographic characteristics of women who visited public health facilities in Mekelle city Tigray, Ethiopia, 2014

Variable – *N* = 367	Frequency	Percent
Age		
15–19	16	4.4
20–24	124	33.7
25–29	148	40.3
30–34	52	14.2
35+	27	7.4
Religion		
Orthodox	317	86.3
Muslim	30	8.2
Catholic	12	3.3
Protestant	8	2.2
Marital Status		
Married	318	86.6
Single	31	8.4
Others^a^	18	5.0
Education		
Illiterate	75	20.4
primary	99	27.0
Secondary	99	27.0
College and above	94	25.6
Occupation		
house wife	232	63.2
Governmental employee	60	16.3
Private employee	27	7.4
Merchant	26	7.1
Daily work	22	6.0
Education of Husband		
Illiterate	42	11.4
primary	92	25.1
Secondary	97	26.4
college and above	136	37.1
Occupation of Husband		
governmental employee	104	28.3
private employee	103	28.1
Merchant	72	19.6
daily work	88	24.0

^a^widowed, separated and divorced

The mean age of the respondents was 26.14 ± 4.67 years. Two hundred fifty six (69.8%) respondents reported that their monthly family income was greater than 1500 Ethiopian Birr followed by 96 (26.2%) with monthly family income 500–1500 Birr and only 15 (4.1%) reported that their monthly family income was less than 500 Birr. The majority 335 (91.3%) of respondents reported that they lived within a walking distance of less than 30 min to the nearest health facility.

### Obstetric history of women

Three hundred sixty two (98.8%) of the respondents reported that their last pregnancy was wanted. Three hundred twenty five (88.6%) of the respondents delivered in health facilities and 322(99.1%) of them had visited ANC at least once during pregnancy. Of the total respondents, 42 (11.4%) had faced complication during delivery (Table 2).

**Table 2:** Obstetric history and knowledge on PNC service of women who visited public health facilities in Mekelle city, Tigray, Ethiopia, 2014

Variables - N = 367	Frequency N	Percent %
Number of pregnancy		
One	141	38.4
Two	121	33.0
Three	61	16.6
Four and above	44	12.0
ANC visit (at least one)		
Yes	344	93.7
No	23	6.3
Frequency (Number) of ANC visit		
< three	17	5.0
Three	53	15.4
Four and above	274	79.6
Months of pregnancy at first visit of ANC		
< four month	115	33.4
At four month	111	32.3
At five month	68	19.8
> Five month	50	14.5
Mode of delivery		
Normal	323	88.0
Caesarean section	22	6.0
Instrumental	22	6.0
Source of information (*N* = 170)		
Health professional	128	75.3
Television	31	18.2
Other^a^	11	6.59
Do you know required frequency of PNC visits (N = 170)		
Yes	73	42.9
No	97	57.1

^a^Friends, neighbors and HEW

### Knowledge of women on PNC services

One hundred seventy (46.3%) of the respondents knew about the availability of PNC service at health facilities. From these, 73 (42.9%) of the respondents said they knew about the frequency of PNC visits (Table 2).

Among those who knew about the presence of PNC services, 57 (33.5%) knew that PNC service is important to get immunization for baby; 48 (28.2%) distinguished that PNC is important to get family planning service; thirty seven (21.8%) recognized PNC is important to prevent health problem of mother and baby arising during delivery and post-delivery besides twenty eight (16.5%) also identified that PNC service is useful to get counseling on feeding practice of baby.

### Practice of PNC by women

Of the 367 respondents, only 118 (32.2%) had visited PNC clinic at least once within 42 days of delivery. From the 118 respondents, 88 (74.6%), 27 (22.9%) and 3(2.5%) had visited once, twice and three or more times respectively. Women who visited within 15–42 days for the first time were 61(51.7%) and women who visited within 7–14 days for the first time were 34 (28.8%) while women who visited within 3 days for the first time were 23 (19.4%) only.

Respondents who had visited ANC at least once 116 (33.7%) had visited PNC clinics. Respondents who knew the availability of PNC service 99 (71.2%) had visited PNC services. Respondents who had faced complications during delivery, 26 (61.9%) had utilized PNC services and almost all 20 (95.2%) respondents who had previous history of obstetrics problems had visited PNC services. Respondents who knew the time of visit of PNC services, 52 (71.2%) had utilized PNC services (Table 3).

**Table 3:** PNC utilization of respondents who visited public health facilities in Mekelle city, Tigray, Ethiopia, 2014

Variable - *N* = 367	Yes = 118	No = 249	Total (367)
	Frequency No (%)	Frequency No (%)	Frequency No (%)
Age			
15–19 year	6(5.1)	10 (4.0)	16 (4.4)
20–24 year	39(33.1)	85(34.1)	124(33.8)
25–29 year	47(39.8)	101(40.6)	148(40.3)
30–34 year	15(12.7)	37(14.9)	52(14.2)
35+	11(9.3)	16(6.4)	27(7.4)
Religion			
Orthodox	99(83.9)	218(87.6)	317(86.4)
Muslim	11(9.3)	19(7.6)	30(8.2)
Catholic	7(5.9)	5(2.0)	12(3.3)
Protestant	1(0.8)	7(2.8)	8(2.2)
Educational status			
Illiterate	11(9.3)	64(25.7)	75(20.4)
Primary	33(28)	66(26.5)	99(27.0)
Secondary	27(22.9)	72(28.9)	99(27.0)
College and above	47(39.8)	47(18.9)	94(25.6)
Marital status			
Married	103(87.3)	215(86.3)	318(86.6)
Single	9(7.6)	22(8.8)	31(8.4)
Separated	1(0.8)	5(2)	6(1.6)
Divorced	1(0.8)	2(0.8)	3(0.8)
Widowed	4(3.4)	5(2)	9(2.5)
Occupation			
House wife	56(47.5)	176(70.7)	232(63.2)
Governmental employee	27(22.9)	33(13.3)	60(16.3)
Private employee	18(15.3)	9(3.6)	27(7.4)
Merchant	13(11)	13(5.2)	26(7.1)
Day laborer	4(3.4)	18(7.2)	22(6.0)
Husbands Education			
Illiterate	10(8.5)	32(12.9)	42(11.4)
Primary Education	21(17.8)	71(28.5)	92(25.1)
Secondary	28(23.7)	69(27.7)	97(26.4)
College and above	59(50)	77(30.9)	136(37.1)
Husband occupation			
Governmental employee	43(36.4)	61(24.5)	104(28.3)
Private employee	29(24.6)	74(29.7)	103(28.1)
Merchant	29(24.6)	43(17.3)	72(19.6)
Day laborer	17(14.4)	71(28.5)	88(24.0)
Number of pregnancy			
One	51(43.2)	90(36.1)	141(38.4)
Two	37(31.4)	84(33.7)	121(33.0)
Three	22(18.6)	39(15.7)	61(16.6)
Four and above	8(6.8)	36(14.5)	44(12.0)
Mode of delivery			
Normal	97(82.2)	226(90.8)	323(88.0)
Instrumental	11(9.3)	11(4.4)	22(6.0)
Caesarean	10(8.5)	12(4.8)	22(6.0)

Ninety two (35.9%), 23(24%) and only 3(20%) of the respondents with monthly family incomes greater than 1500 Birr, between 500 and 1500 birr and less than 500 Birr respectively had utilized PNC services. One hundred thirteen (33.7%) respondents who lived in a distance less than 30 min on foot and 5 (15.6%) respondents who lived greater than 30 min from the health facility had utilized PNC service.

### Respondents’ reasons for visiting health facility

**Fig. 1:**
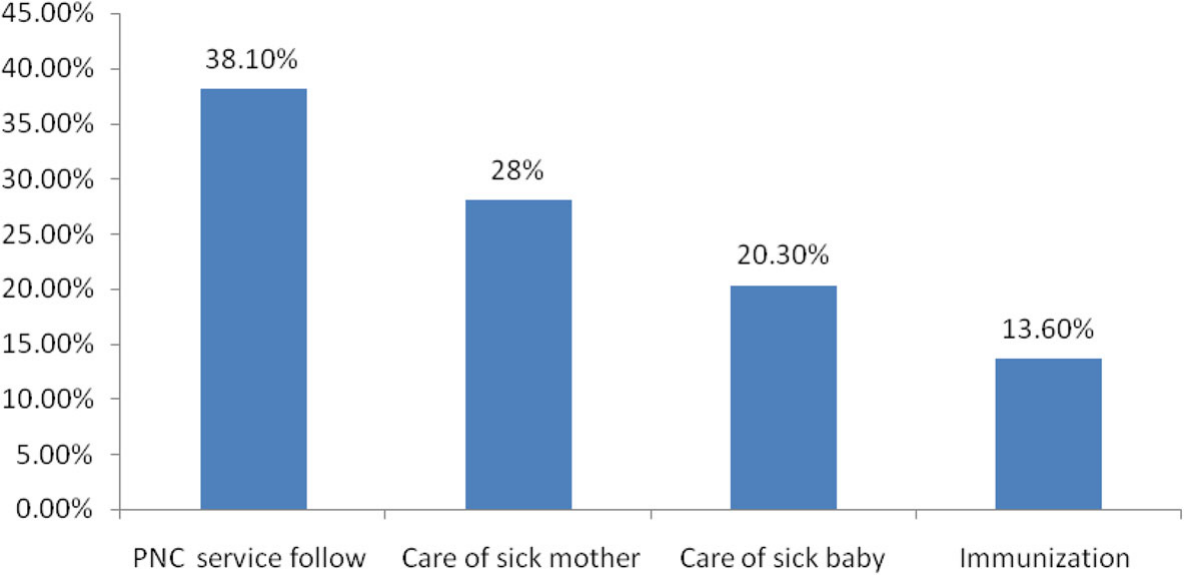
Reasons for visiting PNC clinics of women who visited public health facilities in Mekelle city, Tigray, Ethiopia/2014, *n* = 118

Among women who visited health facility, 45 (38.1%) visited to have a PNC checkup while the rest visited either because they were sick themselves, or their child was sick or to have their child immunized (Fig. 1).

### 5.4.2 Respondents reason for not utilizing of PNC service

**Fig. 2:**
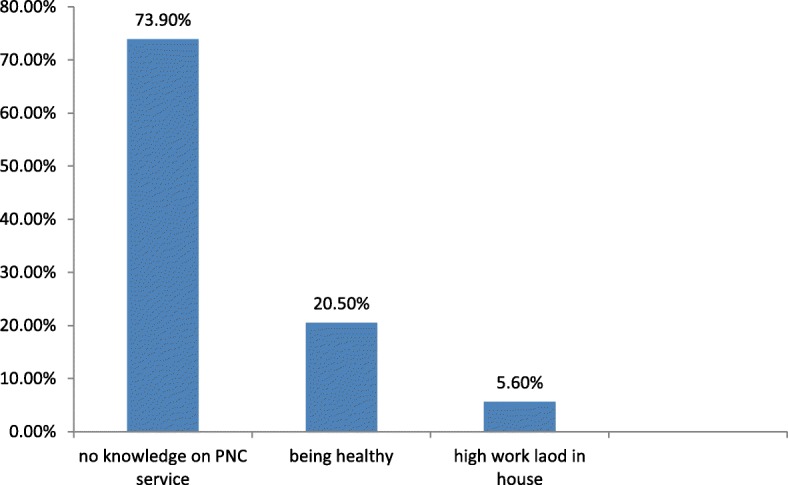
Reasons for not utilizing PNC service of women who visited public health facilities in Mekelle city, Tigray, Ethiopia/2014, *n* = 249

The majority of the respondents 184(73.9%) reported that the reason for not utilizing of postnatal care service was that they did not know the availability of PNC services in the health facilities (Fig. 2).

### Contents of PNC service which women had received during their visit

The report showed that of the women who had visited PNC clinic, most 103 (87.3%) had received less than six elements of postnatal care services; 36 (30.5%) received only two elements and 24 (20.3%) women received three elements of postnatal care service from fourteen elements mentioned above (Table 4).

**Table 4:** Contents of PNC service which women had received during their visit of women who visited public health facilities in Mekelle city, Tigray Ethiopia 2014 *N* = 118

Type of service	Frequency No (%)
Mothers body temperature measurement	57(48.3)
Examination of breast	15(12.7)
Examination for vaginal bleeding	39(33.1)
Mothers blood pressure measurement	21(17.8)
Counseling on exclusive breast feeding	13(11.0)
Counseling on family planning	64(54.2)
Counseling on HIV transmission	6(5.1)
Counseling on care of baby	13(11.0)
Counseling on baby danger signs	22(18.6)
Counseling on personal hygiene	5(4.2)
Immunization of baby	31(26.3)
Checking hygiene of cord	48(40.7)
Measuring body temperature of baby	5(4.2)
Measuring weight of baby	81(68.6)

### Predictor variables of PNC service utilization

Bivariate and multivariate analyses were done to identify independent variables that show significant association for utilization of PNC services. All variables which showed statistically significant association with *p*-value < 0.05 during the bivariate analysis were entered to multivariate analysis and significance was decided at p-value < 0.05 (Table 5).

**Table 5:** Predictor variables of PNC service utilization of women who visit public health facilities in Mekelle city, Tigray, Ethiopia /2014 *n* = 367

	Utilization of PNC service			
Variable	Yes, N(%)	No, N(%)	OR(95% CI)	Adjusted OR(95% CI)
Education				
Illiterate	11(14.7)	64(85.3)	1	1
Primary	33(33.3)	66(66.6)	2.91(1.355, 6.246)*	3.02(0.92, 9.85)
Secondary	27(27.3)	72(72.7	2.18(1.00, 4.75)	1.09(0.31, 3. 89)
College and above	47(50.0)	47(50.0)	5.81(2.73, 12.40)*	1.17(0.28, 4.88)
Occupation				
House wife	56(24.1)	176(75.9)	1	1
Governmental employee	27(45.0)	33(55.0)	2.57(1.42, 4.64)*	1.38(0.56, 3.43)
Private employee	18(66.7)	9(33.3)	6.29(2.67, 14.78)*	6.46(1.91, 21.86)*
Merchant	13(50.0)	13(50.0)	3.14(1.37, 7.17)*	3.34(1.10, 10.19)*
Day laborer	4(18.2)	22(81.8)	0.70(0.23, 2.15)	1.75(0.39, 7.85)
Husband education				
Illiterate	10(23.8)	32(76.2)	1	1
Primary	21(22.8)	71(77.2)	0.95(0.40, 0.22)	0.34(0.08, 1.37)
Secondary	28(28.9)	69(71.1)	1.30(0.56, 2.99)	0.32(0.07, 1.43)
College and above	59(43.4)	77(56.6)	2.452(1.11, 5.39)*	0.49(0.10,2.48)
Husband occupation				
Governmental employee	43(41.3)	61(58.7)	2.94(1.53, 5.68)*	1.36(0.48, 3.87)
Private employee	29(28.2)	74(71.8)	1.64(0.83, 3.24)	0.85(0.33, 2.19)
Merchant	29(40.3)	43(59.7)	2.82(1.39, 5.72)*	1.63(0.62, 4.27)
Day laborer	17(193)	71(80.7)	1	1
Distance				
< 30 minuets	113(33.7)	222(66.3)	2.75(1.03, 7.33)*	1.90(0.55, 6.58)
> 30 minuets	5(15.6)	27(84.4)	1	1
Number of pregnancies				
Four and above	8(18.2)	36(81.8)	1	1
One	51(36.2	90(63.8)	2.55(1.10, 5.90)*	3.35(1.10, 10.21)*
Two	37(30.6)	84(69.4)	1.982(0.840, 4.676)	2.30(0.76, 7.02)
Three	22(36.1)	39(63.9)	2.538(1.004, 6.417)*	2.28(0.69,7.54)
ANC visit				
Yes	116(33.7)	228(66.6)	5.34(1.23, 23.18)*	1.01(0.15, 6.68)
No	2(8.7)	21(91.3)	1	1
Knowledge about availability of PNC				
Yes	99(58.2)	71(41.8)	13.06(7.44, 22.93)*	14.46(7.53, 27.77)*
No	19(9.6)	178(90.4)	1	1

*statically significant at P < 0.05

In bivariate analysis, women’s educational level and occupation, husband’s education and occupation, walking distance from health facility, number of pregnancies, practice of ANC and knowledge PNC service availability showed statistically significant association. In multivariable analysis, women’s occupation, number of pregnancies and knowledge of postnatal care service availability showed statistically significant association on women PNC service utilization (Table 5).

As indicated in Table 5, women’s occupation showed strong association with utilization of postnatal care service utilization. Accordingly, women who were private employees and business women were 6.46 and 3.34 times more likely to utilize postnatal care services (AOR = 6.46, 95% CI: 1.91–21.86) and (3.35, 95% CI: 1.10–10.19) respectively in contrast to unemployed women or house wives. In addition, utilization of PNC services decreased as the number of pregnancies increased. The odds ratio of PNC service utilization of women who had history of one pregnancy was 3.2 times more likely (AOR = 3.19, 95% CI: 1.06–9.57) in contrast to women who had a history of four and more pregnancies.

Knowledge of women of PNC service availability was yet another predictor variable for postnatal care service utilization. As knowledge of women of postnatal care service availability increased utilization of PNC service increased. Women who had knowledge of postnatal care service availability promoted by the health facilities were 14.46 times more likely to utilize postnatal care service (AOR = 14.46, 95% CI: 7.55–27.75) than women who lacked knowledge of the services (Table 5).

## Discussion

The study assessed the level of postnatal care service utilization of women and identified factors that influence utilization of the service in women who visited the public health facilities in Mekelle. The study showed that PNC utilization was low. The main reason reported for not utilizing PNC service was lack of knowledge on the availability of PNC service in public health facilities. According to respondents, the contents of health information on postnatal care services women received were low. The factors that showed significant associations with PNC service utilization were occupation of women, number of pregnancies and knowledge on the availability of PNC services.

Overall, 32.2% women utilized PNC services at least once. Although the study was conducted in women who visited public health facilities for any MCH service, the utilization of postnatal care service was particularly low. The finding was comparable with the findings of EDHS 2011, in which 32% of urban women had utilized the service [2]. However, the finding was higher than a similar study done in north Gondar, Ethiopia where only 6.3% women utilized PNC service [11], another study conducted in Jabitena district, Amhara region where 20.2% women utilized the service [15] and a study done in four regions of Ethiopia (Amhara, Oromia, Southern Nations, Nationalities and People’s Region, and Tigrai) where only 10.6% women utilized PNC services [16]. The possible reasons for the variations might be due methodological differences of the studies and differences in study subject’s residence and the period. However, further investigation is needed to investigate and explain such variation.

The finding of the current study was lower than studies conducted in Sidama zone, southern Ethiopia where 37.2% of the women utilized the PNC services [13] and much lower than a study conducted in Adwa, Tigrai, where 78.3% of the mothers utilized postnatal care services [17]. This difference might be due to the difference in the operational definition of postnatal care service utilization of the studies. The study in Sidama zone, PNC service utilization was considered when the baby had received full immunization; and as the study conducted in Adwa, utilization of the service was considered when women utilized PNC service within 6 months. However, the current study considered women who utilized PNC service within 6 weeks (not months). The current study was also lower in PNC service utilization than a study in Gondar Zuria district where 66.83% women utilized PNC service [18]. Almost half of the respondents in the study in Gondar Zuria, received PNC services from primary health care workers (known locally as health extension workers-HEWs) while the current study considered PNC service utilization provided by skilled health professionals in public health facilities.

The major reason for not attending postnatal care service was lack of knowledge on the availability of PNC services. Although the majority of the respondents had come for ANC and delivered in health facilities, they didn’t know and hear about PNC service availability and provision in health facilities.

Of the respondents who visited PNC services, the majority did so only once. Almost half of the respondents visited within 15–42 days for the first time.. Based on WHO recommendations on postnatal care service of the mother and newborn, mothers should visit for at least four times and the time of first visit should be within 24 h, to be followed on day 3 (48–72 h), between 7 and 14 days after birth, and 6 weeks after birth. The preferred early visits are within 7 days [7]. Nevertheless, findings of the current study indicated that the majority of women did not utilize PNC service as recommended by WHO and were late. Almost all women who had complications during PNC period had visited PNC clinics. This implied that women visit health facilities only when they face complications and in illness.

This study indcated no woman had received complete service. The majority obtained less than five elements out of thirteen mentioned elements of PNC service which are expected to be provided to a mother and new born. Among the components of PNC services, the highest missed opportunity was counseling of exclusive breast feeding where only 12% women had received the information from health providers during their PNC visit. The other element of PNC service with highest missed opportunity was measurement of blood pressure where only 17.8% of women received this service. Similarly, only 6.8% of the babies whose mothers’ visited health facility for PNC had their body temperature measured. This shows that the quality of PNC service provided by public health facilities of the study area is poor when measured in terms of the content of the service given. Thus, training and monitoring health workers to provide a standard PNC service is necessary.

From the socio demographic characteristics of respondents, age,women’s occupation was the predictor variable for utilization of PNC service. Respondents employed on private firms and business women utilized PNC service better compared to unemployed or house wives. The result is similar to a study conducted in Bangladesh [19] where house wives were less likely to utilize PNC service compared to employed women. The result of the current study was also similar to a study conducted in Adwa town, Tigrai, [17] where employed women were more likely to utilize PNC service than women who had no job. This might suggest that women who have their own source of income are more empowered and have a better chance of making decision on seeking health services.

Number of pregnancies was the other factor which was found to be predictor of PNC service utilization. Women who had four and above children were less likely to utilize postnatal care service compared to women who had one child. The result is similar to studies conducted in India [20] and Indonesia [21] where women with two or more children had utilized less postnatal care service than women who had a child for the first time. The current study findings corroborates with studies conducted in southern Ethiopia [13], in Jabitena district, Amhara region [15], and in four regions of Ethiopia (Amhara, Oromia, Southern Nations, Nationalities and People’s Region, and Tigrai) [16], where utilization of postnatal care service was found to decrease as number of pregnancy increase. Women who got four and more pregnancies were less likely to utilize PNC compared to primi gravida women. More difficult labor and complications are believed to occur among women who become pregnant for the first time compared to women with the second and above pregnancies [13].

Knowledge on postnatal care service availability was found as a major factor that affects PNC service utilization. Women who were knowledgeable on PNC service were more likely to utilize PNC service compared with those who did not know. The result corroborates with similar studies conducted in Nepal [22] and Tanzania where women who were aware on PNC service availability were more likely to utilize the service than not [23].

The findings of this study should be interpreted with caution. First, this study was conducted at facility level and institutional-based assessments of barriers to postnatal care may be associated with different barriers than those identified by community-based studies. Secondly, the source of data for this study was based on self-report of respondents and no validation was done with other sources such as cards and registers.

## Conclusion

The finding of this study revealed that utilization of postnatal care service among women who visited public health facilities in Mekelle city was low. Although the majority of women were accessible to public health facilities and the service is provided for free for all women, utilization of the service was low. The main reason for not utilizing the service was lack of knowledge on postnatal care service provided in health facilities. The respondents who received PNC service reported the majority didn’t get a complete service for themselves and their babies.

Further, this study found women’s occupation, number of pregnancies and knowledge on the availability of postnatal care service to be significantly associated with utilization of postnatal care services.

## Additional file

Additional file 1: English Version Questionnaire. (DOC 220 kb)

## Abbreviations

ANC: Antenatal Care
AOR: Adjusted Odds Ratio
EDHS: Ethiopian Demographic Health Survey
HEP: Health Extension Program
MDGs: Millennium Development Goals
PNC: Postnatal Care
SSA: Sub-Saharan Africa
WHO: World Health Organization
