# Supplementary material for: Postnatal care utilization among urban women in northern Ethiopia: cross-sectional survey
Source: BMC Womens Health. 2018 May 30;18:78. doi: 10.1186/s12905-018-0557-5 (PMC5977469; doi:10.1186/s12905-018-0557-5)
Supplement: Supplementary file 1 — English Version Questionnaire. (DOC 220 kb) [file 12905_2018_557_MOESM1_ESM.doc]

**Questionnaire**

**English Version Questionnaire**

**Results of interview questionnaire** 1. Completed 2. Refused 3. Partially completed

Starting Code for each health facility 1. Mekelle hospital **101** 2. Quiha hospital 201 3. Mekelle health center **301** 4. Aynalem health center **401**  5.Adishum dhun health center **501** 6. Lachi health center **601** 7. Kasech health center **701**

| respondents identification  001. Questionnaire Code______  002. Name of Health facility___________________  003. Respondent’s sub city 1. Hadnet 2. Hawelti 3. Semien 4. Ayder 5. Quiha  6. Kedamay weyane 7. Adi-haki 8. Don’t Know  **Instruction: Circle the appropriate answer** provided and where applicable writes the required responses in the spaces provided.  **SECTION 1: SOCIO DEMOGRAPHIC CHARACTERISTICS OF WOMEN** | | | | |
| --- | --- | --- | --- | --- |
| S.N | | Questions | Answers | |
| 101 | | When was your date of child birth? | 1. Day [___|___/___] 2. Don’t Know Day…….  3. Month [__|__] 4. Don’t Know Month | |
| 102 | | Why did you come to this hospital/ health center?  More than one answer is possible  (Don’t mention the choices) | 1. I am sick 2. my baby is sick  3. Immunization of the baby 4. For family planning  5. Circumcision 6. Other specify…………………… | |
| 103 | | In what month and year were you  born?(age of the mother ) | 1. Month [___|___] 2. Don’t Know Month  3. Year [__|__|__|__] 4. Don’t Know Year | |
| 104 | | How old were you on your last birthday? | Age in years [___|___] | |
| 105 | | What is your religion? | 1. Orthodox 2. Muslim 3. Catholic 4. Protestant 5. Other (specify)…………. | |
| 106 | | Are you able to read or write a simple  Sentence? | 1. Yes  2. No_____ skip to 109 | |
| 107 | | Had you ever attend formal school? | 1. Yes 2. No ______ skip to 109 | |
| 108 | | What is the highest grade you completed? | 1. grade [____|___]  2. Technical/vocational certificate  3. University/college diploma  4. University/college degree or Higher | |
| 109 | | What is your present Occupation? | 1. Government employee 2. private employee  3. Merchant 4. Daily work 5. Farmer 6. House wife | |
| 110 | | What is your present marital status? | 1. Married 2. Single 3. Separate 4. Divorce  5. Widowed 6. Un married but living together | |
| 111 | | Is your husband able to read or write a simple Sentence? | 1. Yes 2.No_______ skip to 114 | |
| 112 | | Had your husband ever attend formal school? | 1. Yes 2. No ______ skip to 114 | |
| 113 | | What is the highest grade your husband attended? | 1. Grade [___|___]  2. Technical/vocational certificate  3. University/college diploma  4. University/college degree or Higher | |
| 114 | | What is his present Occupation? | 1. Governmental employee 2. Private employee  3. Merchant 4. Daily work 5. Farmer 6. No work | |
| 115 | | What is your family monthly income? | In birr [_________] | |
| 116 | | How long does it take you to walk to the health facility from your home? | Minuit [­_______] | |
| 117 | | What means of transport do you use to get to the health facility?  (More than one answer is possible) | 1. By foot 2. Public transport (Bus, tax and motorcycle)  3. Ambulance 4. Private vehicle  5. Steretur 6. Others (specify)…………………… | |
| 118 | | Did you get help to come to health facility? | 1. Yes 2. No _____ skip to 201 | |
| 119 | | From whom did you get help? | 1. From my husband 2. From relatives  3. From friends 4. from neighbor  5. Others specify ……………….. | |
| 120 | | What type of help you get? | 1. Transport 2. Money 3. Moral  4. Money and Moral 5. Others specify ……………. | |
| **SECTION 2:** **OBSTETRIC** **CHARACTERISTICS OF WOMEN** | | | | |
| 201 | | How many times pregnant were you?  (including those that did not end with a live  births) | | Number [________] |
| 202 | | How many live births altogether did you have? (total number of live births) | | Number [­­­­­­­­­­­­­­­­­­­­­­­­­­­___________] |
| 203 | | What is your baby’s name? | | Name[____________] |
| 204 | | When you got pregnant for [name] did you want to get pregnant at that time? | | 1. Yes  2. No |
| 205 | | Did you have ANC visit when you were pregnant to [name]? | | 1. Yes 2. No  **______**skip to 211 |
| 206 | | How many times you had visited? | | Number[_________] |
| 207 | | When were the times of your visit? | | 1. [___] months for the first  2. [___] months for the second  3. [ _____] months for the third  4. [______]months for the fourth  5. [______]months for the fifth |
| 208 | | Where was your place of visit?  (More than one answer is possible) | | 1. Health center 2. Governmental hospital  3. Private hospital_______ skip to 211  4. private clinic _______ skip to 211 |
| 209 | | Is this hospital /health center?(if the answer is health center and place of interview is health center, answer is hospital and place of interview is hospital) | | 1. Yes _______skip to 211  2. No |
| 210 | | Why you didn’t go to that hospital/health center? | | 1. The health care providers respect is not good  2. Their waiting time is too long  3. Their service is not complete  4. There is no privacy during examination  5. Their waiting room is not good  6. Others specify ………………….. |
| 211 | | Where did give birth to [name]? | | 1. Home 2. Health center 3. Governmental hospital 4. Private hospital 5. Private clinic |
| 212 | | Who assisted you with the delivery of (name)? | | 1.Health Professional  2. Trained Traditional Birth Attendant  3. Untrained Traditional Birth Attendant  4. Relative/Friend/Neighbor  5. Health extension worker 6. Other, specify________ |
| 213 | | Mode of delivery? | | 1. Normal delivery 2. Instrumental deliver  3. Caesarean section |
| 214 | | Had you faced any complication during the delivery of (name) for yourself/your child? | | 1 Yes 2. No ______skip to 217 |
| 215 | | What type of complication you was faced? | | 1. No 2. Intra partum hemorrhage  3. Postpartum hemorrhage 4. Exlamcia  5. Sepsis 6. Others specify…………………………. |
| 216 | | What type of complication was your baby faced? | | 1. No 2. Underweight 3. Asphyxia  4. Sepsis 5. Others specify……………….. |
| 217 | | After you deliver how long did you stay in the health facility? | | After --------------hours/days |
| 218 | | After you deliver did face any problem for yourself or your child with in 42days? | | 1. Yes 2. No skip to 301 |
| 219 | | What problems you had faced for yourself? | | 1. No 2. Sepsis 3. postpartum hemorrhage  4. Exlamcia 5. Others specify…………………………. |
| 220 | | What problems your baby had faced? | | 1. No 2. Sepsis 3. Infection of cord  4. Others specify…………………… |
| **SECTION 3: KNOWLEDGE OF MOTHERS TOWARDS PNC SERVICE** | | | | |
| 301 | Had you ever heard about post natal care service present in the health facility? | | | 1. Yes 2. No ___________Skip to 401 |
| 302 | From Where did you hear the information?  (More than one answer is possible) | | | 1. From the health professional 2. From friends  3. From relatives 4. From neighbor  5. From health extension workers 6. From radio  7. From .TV 8.Other______________ |
| 303 | Do you know any advantage PNC service? | | | 1. Yes 2. No ______skip to 305  3. I don’t know _______skip to 305 |
| 304 | What are the advantages of PNC service?  (More than one answer is possible)  (Don’t mention choices) | | | 1.To prevent any health problems of the mother related with delivery  2.To prevent any health problems of the baby after delivery  3. To get counseling on feeding practice of baby  4. To get counseling on importance of immunized baby  5. To get counseling on family planning  6. To get counseling on caring and cleanness of baby  7. Others specify…………………….. |
| 305 | Do you know the time of visits for PNC? | | | 1. Yes 2. No _______skip to 308 |
| 306 | How many times should women go to health facility for PNC? | | | Number[________________________] |
| 307 | What are the times of visits? | | | 1. ______for the first visit 2._______ for the second visit  3. ______for the third visit 4. ______for the fourth visit |
| 308 | Do you know that PNC service in governmental health facilities is free? | | | 1. Yes 2. No |
| 309 | During your ANC visits when you were pregnant to (name) did the health care provider told you to visit the health facility for PNC? | | | 1.Yes  2. No |
| 310 | When you deliver (name) did the health care provider told you to visit the health facility for PNC? (For women who delivered in health facility) | | | 1. Yes  2. No |
| **SECTION 4: PRACTICE OF WOMEN ON PNC SERVICE** | | | | |
| 401 | After you deliver (name) did you ever go to health facility for PNC within six weeks? | | | 1. Yes ______skip to 403 2.No |
| 402 | Why you didn’t go to health facility for PNC service?  (More than one answer is possible)  (Don’t mention the choice) | | | 1. Distance to the health facility is too long  2. I heard the waiting time to get service is too long  3. I heard the health professionals respect is not good  4. Lack of money 5. Lack of transportation  6. Lack of awareness 7. I heard their service is not good  8. Culture doesn’t allow to go away from home at this time 9. High work load in the house  10. I don’t know about PNC service in health facilities  11. Others specify……………… |
| 403 | Why did you attend PNC?  (More than one answer is possible)  (Don’t mention the choices) | | | 1. I feel sick after delivery 2. My baby was sick  3. Immunization of the baby 4. For family planning  5. I know PNC follow up is important for me and my baby health 6. Other specify……………… |
| 404 | How many times you visited health facility for PNC? | | | Number [ __________] |
| 405 | At what time were your visits? | | | 1. ………for the first 2. ………for the second  3. ………for the third 4. ………for the fourth  5. ............... for thefifth |
| 406 | Who gave you the service?  (More than one answer is possible) | | | 1. Doctor 2 . Midwife  3. Nurse 4. HEW 5. Others, Specify---------------- |
| 407 | What postnatal care services you was received?  (More than one answer is possible)  (Don’t mention the choice) | | | 1. YES 2. No 8. I do not know  1. Body temperature measurement 1 2 3  2. Breasts examined 1 2 3  3. Examination for abnormal  bleeding 1 2 3  4. Counseling on Exclusive  Breast feeding 1 2 3  5. Blood pressure measurement 1 2 3  6. Family planning services 1 2 3  7. Counseling about HIV transmission 1 2 3  8. Counseling about care of the baby 1 2 3  9. Counseling on baby danger signs 1 2 3  10. Counseling on personal hygiene 1 2 3  11. Other specify………………… |
| 408 | What postnatal care services did you receive for your baby?  (More than one answer is possible)  (Don’t mention the choice) | | | 1. Yes 2. No 8. I do not know  1. Immunization 1 2 3  2. Checking hygiene of cored and skin 1 2 3  3. Body temperature measurement 1 2 3  4. Checking of the body weigh 1 2 3  5. Other specify………………………………… |
| 409 | Are there problems that you are facing that can prevent you from going health facility to receive PNC services? | | | 1.Yes  2. NO |
| 410 | Can you mention them?  (More than one answer is possible)  (Don’t mention the choice) | | | 1. Distance to the health facility is too long  2. Lack of money 3. Lack of transportation  4. High work load in the house  5. Culture doesn’t allow to go away from home at this time 6. Others specify……………….……………… |

Thank you for participation

**ትግርኛ ቃለ ምሕትት:**

**ሓበሬታና ስምምዕነት ቅጥዒ**

ጥዕናይሃበለይኣነኣይተ/ወ/ሮ________________________ይብሃል::አነ ብዛዕባ ክትትልንን ክንክንን ድሕሪ ወሊድ ግልጋሎት አጠቃቅማ ንዘለው ፀገማትን ዝምልከት ሓበሬታ ክስብስብ እየ መፂአ::እዚ መፅናዕቲ ብወ/ት ገነት ገብረሂዎት ካብ መቐለ ዩኒቨርስቲ ኮሎጅ ጥዕናሳይንስ ክፍሊ ሕብረተሰብ ጥዕና ናይ ካልአይ ዲግሪ ተምሃሪት ኢዩ ዝካየድ ዘሎ፡፡ዕላማ ናይዚ መፅናዕቲ እዚ ምልካዕ ክትትልን ክንክንን ድሕሪ ወሊድ ግልጋሎት ተጠቀምቲ ዝኮና አዴታትን ነዚ ግልጋሎት ዘዕንቅፉ /ዝፀልው ኩነታት ንምንፃርን ምውሳን ዝዓለመ ኮይኑ አብ መቐለ ከተማ ይካየድ አሎ:: አብዚ መፅናዕቲ ንክትሳተፋ ይሓተክን አለኩ :: ንሃትክን ትሕብብር አብ ምፍላይ ንክትትልን ክንክን ድሕረ ወሊድ ግልጋሎት ዘዐንቅፉ ፀገማት ብጣዐሚ ጠቃሚ አዩ:: እቲ መፅናዕቲ ብናይ ፅሑፍ መሕተት እዩ ክካየድ:: ንሓፂር ግዜ ማለት ንኸባቢ 15 ደቒቓ እየ ክሓተክን:: ስምክን አብዚ ፎሚ አይፀሓፍን እና ምስ እትህባና ሓበሬታ አይተሓሓዝን :: አብዚ ብምስታፍክን ምንም ዝበፅሐክን ፀገም የለን :: ኩሉ እትህባና ሓበሬታ ምስጢራዊነቱ ዝተሓለወ እዩ:: ምስታፍክን ብድሌት ዝተመስረተ እዩ ምምላስ ዘይትደልዮኦ ሕቶ ንክትምልሳ አይትግደዳን:: ክትምልስኦ ዘይትደልየኦ ጥያቄ እንተሃልዩ ክትሓልፍኦ መብትክን እዩ:: ንዝመፅናዕቲ ዝተመልከተ ሕቶ እንተሃልዩክን ወይ ውፅኢት ናይዚ መፅናዕቲ ክትፈልጣ እንተደሊክን ብነፃነት ንመቐለ ዩኒቨርስቲ ሕብረተሰብ ጥዕና ስልኪ ቁፅር+251-344-416683, ኢሜል:

እናመፅናቲ እተካይድ ዘላ ወ/ሪት ገነት ገብረሂዎት ብስልኪ ቁፅሪ 251 911095380, ወይ ብኢሜል: [genimeb@gmail.com](mailto:genimeb@gmail.com) ክትሓታ ትክእላ ኢክን::

ንክትሳተፋ ፍካደኛ ዲክን?

1. እወ ናብ ዝቅፅል ገፅይ ሕለፉ

2. አይፋሉን ናብ ዝቅፅል ተሓታቲ ሕለፉ

**ስምምዕነት ቅጥዒ**

አብዚ መፅናዕቲ ንክሳተፍ ፍቀደኛ ምዃነይ እንትገልፅ ናይቲ መፅናዕቲ ዓላማ ምልካዕ ክትትልን ክንክን

ድሕሪ ወሊድ ግልጋሎት ተጠቀምቲ ዝኮና አዴታትን ነዚ ግልጋሎት ዘዕንቅፉ /ዝፀልው ኩነታትን ምንፃርን

ምውሳን ዝዓለመ ከምዝኮነ እና ዝህቦ መልሲ ንካሊእ ጥቅሚ ከምዘይውዕል አብ ዝኮነ ይኩን ቦታ ስመይ ከም

ዘይግልፅ እንደገና ምስታፈይ ብድሌት ዝተመስረተ ምዃኑ እንደገና ክምልሶ ዘይደሊ ሕቶ ክገድፎ ከምዝክእል እና ብምስታፈይ ምንም ፀገም ከምዘይበፅሐኒ ተረዲኤ እየ::

ናይ ተሓታቲ ፊርማ _____________________ ዕለት__________________

ናይ ሓታታይ ስም______________________ፊርማ_________ ዕለት________

ናይ ተዐዓዛባይ ስም__________________ፊርማ______________________

**ውፅኢት ናይ ቃለ መሕትት**

1. ዝተማለአ 2. ዘይተማለአ 3. ብከፊል ዝተማለአ

መጀመሪኮደ 1. መቐለ ሆሰፒታል101 2.ኺሓ ሆሰፒታ 201 3.መቐለ ጥዕና ጣብያ 301

4. አይናለም ጥዕና ጣብያ 401 5. ዓዲ-ሹምድሑን ጥዕና ጣብያ501 6.ላጪ ጥዕና ጣብያ601 7.ካሰች ጥዕና ጣብያ 701

**ቃለመሕተትተጠቃምነትክትትልንክንክንነድሕሪወሊድግልጋሎት**

| 1. ናይ ተሓታቲ መፍለዪ ነጥብታት  001. ናይ ቃለ መሕትት ቁፅሪ……………………  002. ናይ ጥዕና ትካል ስም………………………  003. ናይ ተሓታቲ ክፍለ ከተማ 1.ሓድነት 2. ሓወልቲ 3. ሰሜን 4. ዓይደር 5. ኺሓ 6. ቀዳማይወያነ 7. ዓዲሓቂ 8. አይፈልጦን  **መምርሒ**:ካብ እዞም ኣብ ታሕቲ ዝተዘርዘሩ ሕቶታት በቲ ተሓታቲ ዝተመለሱ መልሲታት ብትኽክል የኽብቡ ብተወሳኺ ኣበቲ ክፍቲ ቦታ ትክክለኛ መልሲ ይፅሓፉ::  **ክፍሊ ሓደ : መሰረታዊ ናይ ኣነባብራ ሓበሬታ** | | | |
| --- | --- | --- | --- |
| ተ.ቁ | ሕቶታት | | መልሰታት |
| 101 | ዝወለድክናሉ ዕለት? | | 1. መዓልቲ [___________] 2. መዓልቱ አይፈልጦን  3. ወርሒ [___________] 4. ወርሒ አይፈልጦን |
| 102 | ናብዚ ሆስፒታል / ጥዕና ጣብያ እንታይ ክትገብራ መፂክን?  (ካብ ሓደ መልሲ ንላዕሊ ምምላስ ይከአልእዩ)  (መማረፂታት አይነበቡ) | | 1. ስለ ዘሕመመኒ 2. ህፃነይ ስለ ዝሓመመኒ  3. ህፃነይ ከክትብ 4. መከላከሊ ጥንሲ ክክስድ  5. ህፃነይ ከክትብን መከላከሊ ጥንሲ ክክስድን  6. ካሊእ እንተሃልዩ ይገለፅ­­­­­………… |
| 103 | ብመአዝ ወርሒን ዓ/ምን ተወሊድክን?  (ናይ አዶ ዕድመ) | | 1. ወርሒ [___________] 2. ወርሒ አይፈልጦን  3. ዓ/ም [__________________]. 4. ዓ/ም አይፈልጦን |
| 104 | ዕደመኽን ክንደይ ትገብራ?  (ሙሉእ ዓመት ይፀሓፍ) | | ዕድመ ብዓመት [___________]. |
| 105 | ናይ ኣየናይ ሃይማኖት ተኸታሊት ኢኽን? | | 1. ኦርትዶክስ ክርስትያን 2. ሞስሊም  3. ካቶሊክ 4. ፐሮቲሰታንሰ 5. ካሊእ እንተሃልዩ ይገለፅ............... |
| 106 | ክተምብባ ወይ ክትፅሕፋ ትክእላዶ? | | 1. እወ 2. አይፋሉን _______ናብ ቁፅሪ 109 ይዝለላ |
| 107 | ስሩዕ ትምህርቲ ተማሂርክንዶ? | | 1. እወ 2. አይፋሉን _________ናብ ቁፅሪ 109 ይዝለላ |
| 108 | ዝለዓለ ዝበፃሕክንኦ ናይ ትምህርቲ ደረጃ? | | 1. ክፍሊ [_________] 2. ሰርተፊኬት  3. ኮለጅ / ዩኒቨርስቲ ዲፕሎማ 4. ኮለጅ / ዩኒቨርስቲ ዲግሪ |
| 109 | ሐዚ ዘለኽነኦ ኩነታት ስራሕ እንታይ እዩ? | | 1. ናይ መንግስቲ ቁፃር 2. ናይ ዉልቀ ቁፃር 3. ነጋዴ  4. መዓልታ ዊስራሕ 5. አብ ገዛ እትውዕል |
| 110 | ኩነታት ሓዳር? | | 1. ዝተመርዓወት 2. ዘይተመርዐወት 3. ዝተፋተሐት  4. ብዓል ገዝአ ዝሞታ 5. ተፈላልያ እትነብር  6. ዘይተመርዐወት ግን ሓቢራ እትነብር |
| 111 | በዓል ገዛክን ከምብቡ ወይ ክፅሕፉ ይክእሉዶ? | | 1. እወ 2. አይፋሉን _________ናብ ቁፅሪ 114 ይዝለላ |
| 112 | በዓል ገዛክን ስሩዕ ትምህርቲ ተማሂሮምዶ? | | 1. እወ 2. አይፋሉን __________ናብ ቁፅሪ 114 ይዝለላ |
| 113 | ዝለዓለ ዝበፅሕዎ ናይ ትምህርቲ ደረጃ? | | 1. ክፍሊ [_________] 2. ሰርተፊኬት  3. ኮለጅ / ዩኒቨርስቲ ዲፕሎማ 4. ኮለጅ / ዩኒቨርስ ቲዲግሪ |
| 114 | በዓል ገዛክን ሕዚ ዘለዎም ናይ ስራሕ ኩነታት እንታይ እዩ | | 1. መንግስታዊ ቁፃር 2. ናይ ዉልቀ ቁፃር 3. ነጋዴ  4. መዓልታ ዊስራሕ 5. ስራሕ ዘየብሎም |
| 115 | ናይ ቤተሰብክን ወርሓዊ አታዊ ክንደይ ይከውን? | | ብቅርሺ [__________] |
| 116 | ካብ ገዛክን ናብ ጥዕና ትካል ብእግርክን ንክትከዳ ክንደይ ዝአክል ግዜ ይወስደልክን? | | ብደቒቓ [______________________] |
| 117 | ናብ ጥዕና ትካል ንክትከዳ እንታይ ዓይነት መጓዓዝያ ትጥቀማ?  (ካብ ሓደ መልሲን ላዕሊ ምምላስ ይከአል እዩ) | | 1. ብእግረይ  2. ብናይ ህዝቢ መጓዓዝያ (ኣውቶቡስ,ታክሲ, ሞተር ሳይክ ወዘተ)  3. ኣምቡላንሰ 4. ብናይ ግለይ መጓዓዝያ  5. ቃረሬዛ /ባህላዊ ኣምቡላንሰ 6. ካሊእ (ይገለፅ)............................ |
| 118 | ናብ ጥዕና ትካል ንክትመፃ ዝኮነ ዓይነት ሓገዝዶ ረኪብክን ነይርክን? | | 1. እወ 2. አይፋሉን________ናብ ቁፅሪ 201 ይዝለላ |
| 119 | ካብ መን ኢክን ሓገዝ ረኪብክን?  (ካብ ሓደ መልሲ ንላዕሊ ምምላስ ይከአል እዩ) | | 1. ካብ ብዓል ገዛይ 2. ካብ ቤተሰብ 3. ካብ መሓዛይ  4. ካብ ጎረቤተይ 5. ካሊእ እንተሃልዩ ይገለፅ…………………. |
| 120 | እንታይ ሓገዝ ኢክን ረኪብክን?  (ካብ ሓደ መልሲ ንላዕሊ ምምላስ ይከአል እዩ) | | 1. ናይ መጓዝያ 2. ናይ ገንዘብ 3. ናይ ሓሳብ  4. ናይ ገንዘብንሓሳብ 5.ካሊእ እንተሃልዩ ይገለፅ........................... |
| **ክፍሊክልተ፡ ኩነታት ሕሉፍ በዝሒ ጥንስን ውላድን** | | | |
| 201 | ክንደይ ግዜ ዝኣክል ጠኒስክን ነይርክን?  (ብሂዎት ዘይተመወለዱ እውን ሓዊስካ) | | በዝሒ ብቁፅሪ [____________] |
| 202 | ጠቅላላ ብሂወቶም ዝተወለዱ ቆሉዕክን ክንደይ ይኮኑ? | | በዝሒ ብቁፅሪ [___________] |
| 203 | ቆልዓክን ስማ/ሙ መን ትበሃል/ይበሃል? | | ስም [_____________] |
| 204 | ን……..(ስም) ክትጠንሳ ከለክን ደሊክንኦ ድዩ ወይስ ብአጋጣሚ ኢክን ጠኒስክን? | | 1. እወ  2. አይፋሉን |
| 205 | ን…….(ስም) ጥንስቲ እንተለክን ክትትልን ክንክንን ቅድመ ወሊድዶ ትገብራ ነይርክን? | | 1. እወ  2. አይፋሉን ________ናብ ቁፅሪ 211 ይዝለላ |
| 206 | ንክደይ ግዜ ክትትል ጌርክን? | | በዝሒ ብቁፅሪ [___________] |
| 207 | ዝተከታተልክናሉ ግዜ መአዝ መአዝ ነይሩ? | | 1. ….­­­­­­­­­­­­­­­­­­­­­­­­ ወርሒ ንመጀመርያ ግዜ 2……ወርሒ ንካልኣይ ግዜ  3. ….ወርሒ ንሳልሳይ ግዜ 4. ….ወርሒ ንራብዓይ ግዜ  5. ………………ወርሒ ንሓምሻይ ግዜ |
| 208 | አበይ ኢክን ትከታተላ ነይርክን?  (ካብ ሓደ መልሲ ንላዕሊ ምምላስ ይከአል እዩ) | | 1. አብ ናይ መንግስቲ ጥዕና ጣብያ 2. አብ ናይ መንግስቲ ሆስፒታል  3. አብ ናይ ዉልቀ ሆስፒታል _______ናብ ቁፅሪ 211 ይዝለላ  4. አብ ናይ ውልቀ ክሊኒክ _________ናብ ቁፅሪ 211 ይዝለላ |
| 209 | አብዚ ሆሰፒታል / ጥዕና ጣብያ ድዩ ወይስ አብ ካሊእ ሆሰፒታል / ጥዕና ጣብያ?  (መልሰን እና ዝጥየቃሉ ዘለዋ ቦታ ሆስፒታል እንተኮይኑ /መልሰን እና ዝጥየቃሉ ዘለዋ ቦታ ጥዕና ጣብያ እንተኮይኑ) | | 1. እወ________ናብ ቁፅሪ 211 ይዝለላ  2. አይፋሉን |
| 210 | ንምንታይ ኢይክን ናብቲ ሆሰፒታል / ጥዕና ጣብያ ዘይከድክን?  (ካብ ሓደ መልሲ ንላዕሊ ምምላስ ይከአል እዩ)  (መማረፂታት አይነበቡ) | | 1.እቶም ጥዕና ሰብ ሞያታት ስለ ዘየክብሩ  2. ንነዊሕ ግዜ እዮም ዘፀብዩ  3. ምሉእ አገልግት ስለ ዘይህቡ  4. ናይ ውልቀ ዝኮነ መመርመሪ ክፍሊ ስለ ዘየብሎም  5. መፀበይ ቦትኦም ፅቡቅ አይኮነን  6. ካሊእ እንተሃልዩ ይገለፅ……………………… |
| 211 | ን……..(ስም) አበይ ወሊድክንአ/ኦ? | | 1. አብ ገዛ 2. አብ ናይ መንግስቲ ጥዕና ጣብያ  3. አብ ናይ መንግስቲ ሆስፒታል 4. አብ ናይ ዉልቀ ሆስፒታል  5. አብ ናይ ውልቀ ክሊኒክ |
| 212 | ን…….(ስም) ክትወልዳ ከለክን ብመን ተሓጊዝክን? | | 1. ብጥዕና ሰብሞያ  2. ብዝሰልጠና ባህላዊ መዋለድቲ  3. ብዘይሰልጠና ባህላዊ መዋለድቲ  4. ብቤተሰብ/ መሓዛይ / ጎረቤት  5. ብናይ ጥዕና ፓኬጅ ሰራሕተኛ  6. ካልእ እንተሃልዩ ይገለፅ………………………. |
| 213 | ዓይነት /ኩነታት ሕርሲ? | | 1. ብስሩዕ ወይ ንቡር ሕርሲ  2. ብሓገዝ መሳርሒ/ ቫክዩም፣ብፎርሴፍ ስወዘተ/ 3.ብቀይሳራዊ መጥባሕቲ |
| 214 | ን…….(ስም) ክትወልዳ ከለክን አብ ባዕልክን ኮነ ኣብ ቆልዓክን ዝኮነ ፀገም ኣጋጢሙኩም ነይሩዶ? | | 1. እወ 2. አይፋሉን _________ናብ ቁፅሪ 217 ይዝለላ |
| 215 | ንስክን እንታይ ዓይነት ፀገምአጋ ጢሙክን ነይሩ?  (ካብ ሓደ መልሲ ንላዕሊ ምምላስ ይከአል እዩ)  (መማረፂታት አይነበቡ) | | 1. አብ እዋን ሕርሲ ብርቱዕ ናይ ማህፀን ደም ምፍሳስ  2. ድሕሪ ወሊድ ብርቱዕ ናይ ማህፀን ደም ምፍሳስ  3. ናይ ደም መጠን ምውሳክ 4. ረክሲ 5. ካልእ እንተሃልዩ ይገለፅ……. |
| 216 | ንህፃንክን እንታይ ዓይነት ፀገም አጋጢዎ ነይሩ?  (ካብ ሓደ መልሲ ንላዕሊ ምምላስ ይከአልእዩ)  (መማረፂታት አይነበቡ) | | 1. ትሕቲ ክብደት ተወሊዱ 2. ምዕፋን  3. ረክሲ 4. ካልእ እንተሃልዩ ይገለፅ………………………. |
| 217 | ምስወለድክን ካብ ጥዕና ትካል መአዝ ወፂእክን? | | ብሰዓት/መዓልቲ------------ገፍረሐሐሀ |
| 218 | ድሕሪ ምውላድክን አብ ባዕልክን ኮነ አብ ህንክነ ዘጋጠመክን ናይ ጥዕና ገም ነይሩ ዶ? | | 1. እወ 2. አይፋሉን |
| 219 | እንታይ ዓይነት ፀገም አጋጢመክን ነይሩ? | | 1. አየጋጠመንን 2. ረክሲ 3. ብርቱዕ ናይ ማህፀን ደም ምፍሳስ  4. ናይ ደምመጠን ምወሳክ 5. ካልእ እንተሃልዩ ይገለፅ……………… |
| 220 | ንህፃንክን እንታይ ዓይነት ፀገም አጋጢዎ ነይሩ?  (ካብ ሓደ መልሲ ንላዕሊ ምምላስ ይከአልእዩ)  (መማረፂታት አይነበቡ) | | 1. አየጋጠመንን 2. ረክሲ 3. ረክሲ ናይ ዕትብቲ  4. ካልእ እንተሃልዩ ይገለፅ………………………. |
| **ክፍሊሰለስተ: ኣዴታት ኣብ ክትትልን ክንክንን ድሕሪ ወሊድ ግልጋሎት ዘለወን ፍልጠት** | | | |
| 301 | ብዛዕባ ክትትልን ክንክንን ድሕሪ ወሊድ ግልጋሎት ኣብ ጥዕና ትካል ዝወሃብ ምኻኑ ሰሚዕክንዶ ትፈልጣ/ ? | 1. እወ 2.ኣይፋልን__________ናብ ቁፅሪ 401 ይዝለላ | |
| 302 | ብዛዕባ ክትትልን ክንክንን ድሕሪ ወሊድ ግልጋሎት ኣብ ጥዕና ትካል ዝወሃብ ምኻኑ ካበይ ሰሚዕክን?  (ካብ ሓደ መልሲ ንላዕሊ ምምላሰ ይከአል እዩ) | 1. ካብ ጥዕና ሰብ ሞያታት 2. ካብ ቤተሰብ 3. ካብ መሓዙተይ  4. ካብ ጎረቤት 5. ካብ ጥዕና ፓከጅ ሰራሕተኛታት 6. ካብ ራድዮ  7. ካብ ተለቭዥን 8. ካልእ (ይገለፅ)...................... | |
| 303 | ጥቅሚ ናይ ክትትልን ክንክንን ድሕሪ ወሊድ ትፈልጣዶ? | 1. እወ 2. አይፋሉን _______ናብ ቁፅሪ 305 ይዝለላ  8. አይፈልጥን _______ናብ ቁፅሪ 305 ይዝለላ | |
| 304 | እንታይ ዓይነት ጥቅሚ ትፈልጣ?  (ካብ ሓደ መልሲ ንላዕሊ ምምላሰ ይከአልእዩ)  (መማረፂታት አይነበቡ) | 1. ምስ ወሊድ ዝተተሓሓዘ አብ አዶ ናይ ጥዕና ፀገም ከይፍጠር ንምክልካል  2. አብ ጥዕና ህፃን ፀገም ከይፍጠር ንምክልካል  3. አብ አመጋግባ ህፃን ምክሪ ንምርካብ  4. ንህፃን ምክታብ ዘለዎ ጥቅሚ ምክሪ ንምርካብ  5. አብ ምጣነ ስድራ ንክሪ ንምርካብ  6. አብ አተሓሕዛን ፅሬትን ህፃን ምክሪን ምርካብ  7. ካልእ (ይገለፅ)...................... | |
| 305 | ክትትልን ክንክንን ድሕሪ ወሊድ መአዝ መአዝ ምዃኑ ትፈልጣዶ? | 1. እወ  2. አይፋሉን ______ናብ ቁፅሪ 308 ይዝለላ | |
| 306 | ሓንቲ አዶ ንክንደይ ግዜ ክትከታተል አለዋ ትብላ? | ብቁፅሪ [___________] | |
| 307 | እቶም ናይ ክትትል ግዚያት መአዝ መአዝ እዮም ትብላ? | 1. ….­­­­­­­­­­­­­­­­­­­­­­­­...... ንመጀመርያ ግዜ 2………..ንካልኣይ ግዜ  3………..ንሳልሳይ ግዜ 4. ………ንራብዓይ ግዜ | |
| 308 | ክትትልን ክንክንን ድሕሪ ወሊድ ኣብ ናይ መንግስቲ ጥዕና ትካል ብናፃ ምዃኑ ትፈልጣዶ? | 1. እወ  2. አይፋሉን | |
| 309 | ን ……..(ስም) ጥንስቲ ኮይንክን ቅድመ ወሊድ ክትትል አብ እትገብራሉ ግዜ እቶም ጥዕና ሰብ ሞያታት ድሕሪ ወሊድ ክትትልን ክትገብራ ሓቢሮሙልክንዶ ነይሮም? | 1. እወ  2. አይፋሉን | |
| 310 | ን ……ስም ……አብ እትወልዳሉ ግዜ እቶም ዝሓገዙክን ጥዕና ሰብሞያታት ድሕሪ ወሊድ ክትትል ንክትገብራዶ ሓቢሮሙልክን ነይሮም?  (አብ ጥዕና ትካል ንዝወለዳ) | 1. እወ  2. አይፋሉን | |
| **ክፍሊ አርባዕተ፡ አጠቓቕማ አዴታት አብ ድሕሪ ወሊድ ግልጋሎት** | | | |
| 401 | ድሕሪ ምውላድክን ኣብ 6 ሰሙን ውሽጢ ክትትልን ክንክንን ድሕሪ ወሊድ ግልጋሎት ኣብ ጥዕና ትካል ጌርክንዶ ነይርክን? | 1. እወ________________ናብ ቁፅሪ 403 ይዝለላ  2. ኣይፋልን | |
| 402 | ንምንታይ እክን ክትትልን ክንክንን ድሕሪ ወሊድ ግልጋሎት ዘይገበርክን?  (ካብ ሓደ መልሲ ንላዕሊ ምምላስ ይከአልዩ)  (መማረፂታት አይነበቡ) | 1. ጥዕና ትካል ካብ ገዛይ ሩሑቅ ስለ ዝኮነ  2. ንነዉሕ ግዜ እዮም ዘፀብዩ ስለ ዝሰማዕኩ  3. እቶም ጥዕና ሰብሞያታት አየክብሩዃን ስለ ዝሰማዕኩ  4. ገንዘብ ስለ ዝሰአንኩ  5. መጓዝያ ስለ ዝሰአንኩ  6. ናይ ግንዛቤ ሕፅረት ስለ ዝነበረኒ  7. ምሉእ አገልግሎት አይህቡን ዝሰማዕኩ  8. ብባህልና አብቲ ግዜ እቲ ካብ ገዛ ምውፃእ ስለ ዘይፍቀድ  9. ናይ ገዛ ስራሕ ስለ ዝበዝሐኒ  10 ኸትትል ድሕሪ ወሊድ አብ ጥዕና ትካል ከምዝወሃብ አይፈልጥን  11. ካሊእ እንተሃልዩ ይገለፅ…………….... | |
| 403 | ንምንታይ ኢክን ናብክትትልን ክንክንን ድሕሪ ወሊድ ኬድክን?  (ካብ ሓደ መልሲ ንላዕሊ ምምላስ ይከአልዩ)  (መማረፂታት አይነበቡ) | 1. ድሕሪ ምውላደይ ስለ ዘሕመኒ  2. እቲ ህፃን ስለ ዝሓመመኒ  3. ህፃን ከክትብ  4. መከላከሊ ጥንሲ ክወስድ  5. ህፃን ከክትብን መከላከሊ ጥንሲ ክወስድን  6. ናይ ድሕሪ ወሊድ ክትትልን ጥዕናይን ጥዕና ህፃነይን ጠቃሚ ስለዝኮነ  7. ካሊእ እንተሃልዩ ይግለፅ………………... | |
| 404 | ንክንደይ ጊዜ ኢኪን ክትትል ጌርክን ? | ብቁፅሪ [________] | |
| 405 | ክትትል ዝገበርክናሉ ግዜ መአዝ መአዝ ነይሩ? | 1. ….­­­­­­­­­­­­­­­­­­­­­­­­........ንመጀመርያ ግዜ 2…………ንካልኣይ ግዜ  3…………ንሳልሳይ ግዜ 4. ……….ንራብዓይ ግዜ  5. ………………ሸንሓምሻይ ግዜ | |
| 406 | እቲ ግልጋሎት ብመን ተዋሂቡክን?  (ካብ ሓደ መልሲ ንላዕሊ ምምላስ ይከአልዩ) | 1. ብዶክተር 2. ብመወዋልዳን 3. ብነርስ 4. ብጥና ፓከጅ ሰራሕተኛ  5. ካሊእ እንተሃልዩ ይግለፅ…………… | |
| 407 | እንታይ ዓይነት አገልግሎት ረኪብክን?  (ካብ ሓደ መልሲ ንላዕሊ ምምላስ ይከአልዩ)  (መማረፂታት አይነበቡ) | 1. እወ 2. አይፋሉን 3. አይፈለጥኩን  1. ሙቐት ናይ ሰውነት ምልካዕ 1 2 3  2. ናይ ጡብ ምርመራ 1 2 3  3. ምርመራ ብርቱዕ ናይ ማህፀን መድመይቲ 1 2 3  4. ምኽሪ ብዛዕባ ፀባ ጡብ ኣዶ ጥራሕ ምምጋብ 1 2 3  5. ምኽሪ ብዛዕባ ምጣነ ስድራ 1 2 3  6. ናይ ደም መጠን ምልካዕ 1 2 3  7. ምኽሪ ብዛዕባ ምምሕል ላፍኤች አይቪ 1 2 3  8. ምክሪ ብዛዕባ ክንክን ህፃን 1 2 3  9. ምክሪ ብዛዕባ ሓደገኛ ምልክታት ህፃን 1 2 3  10. ምክሪ ብዛዕባ ውልቀ ፅሬት 1 2 3  11.ካሊእ እንተሃልዩ ይገለፅ...................... | |
| 408 | ንህፃንክን እንታይ ዓይነት አገልግሎት ረኪብክን?  (ካብ ሓደ መልሲ ንላዕሊ ምምላስ ይከአልዩ)  (መማረፂታት አይነበቡ) | 1.እወ 2. አይፋሉንቨ 3. አይፈለጥኩን  1. ክትባት 1 2 3  2. ሙቐት ናይ ሰውነት ምልካዕ 1 2 3  3. ምርመራ ኩነታት ፅሬት ዕትብቲ 1 2 3  4. ናይ ሰውነት ክብደት ምልካዕ 1 2 3  5. ካሊእ እንተሃልዩ ይገለፅ................ | |
| 409 | ናብ ጥዕና ትካል ንድሕሪ ወሊድ ክትትል ክትከዳ ክትሓስባ ከለክን ዕንቅፋት ዝኮኑክን ነገራት አለውዶ? | 1. እወ 2. አይፋሉን | |
| 410 | እንታይ ዓይነት ነገራት እዮም?  (ካብ ሓደ መልሲ ንላዕሊ ምምላስይ ከአል እዩ)  (መማረፂታት አይነበቡ) | 1. ጥዕና ትካል ካብ ገዛይ ሩሑቅ ምዃኑ  2. ገንዘብ ስለ ዝስእን  3. መጓዝያ ስለ ዝስእን  4. ናይ ገዛ ስራሕ ስለ ዝበዝሐኒ  5. ብባህልና አብቲ ግዜ እቲ ካብ ገዛ ምውፃእ ስለ ዘይፍቀድ  6. ካሊእ እንተሃልዩ ይገለፅ................ | |

ኣብዚ መፅናዕቲ ዚ ብምስታፍክንን ንዝሃብክናና መልሲን ርኢቶን ብጣዕሚ

ነመስግን!!
